# Mr. Veiten, on Vaccine Inoculation

**Published:** 1800-10

**Authors:** James Veiten

**Affiliations:** Edgecombe-street Stonehouse


### *To the Editors of the Medical and Phyſical Journal.*

Gentlemen,

It will give me great pleaſure if the encloſed letter, originally tranſmitted to the Board for Sick and Wounded Seamen is inſerted in your valuable Journal. I am fully aware that it contains little that can be conſidered in any degree new; but it may anſwer the purpoſe of exciting a more extended attention to Vaccine Inoculation amongſt that part of my profeſſion attached to the Navy, as I am myſelf, from indiſpoſition, at preſent perſonally incapable of proſecuting the object my letter holds up to view. A copy of it was ſent to Lord St. Vincent, in conſequence of the Magnificent having ſome communication with the Cumberland, on board of which ſhip the Small-pox exiſted; and the reception it met with from his Lordſhip, proves him to be fully as capable of promoting thoſe branches of ſcience which have a tendency to meliorate the condition of man, in honouring with his attention uſeful ſuggeſtion, as of repreſſing the power of his enemy. But notwithſtanding his Lordſhip's decided approbation, local circumſtances, connected with the Magnificent, wreſted from my hands the pleaſure of actually introducing Vaccine Inoculation into the Navy.

I am, Gentlemen,

Your obedient humble ſervant,

*Edgecombe-ſtreet Stonehouſe, 22 Auguſt, 1800.* JAMES VEITEN.

---

To the COMMISSIONERS for SICK and WOUNDED SEAMEN.

GENTLEMEN,

THE Vaccine Inoculation has, ſince my return from the Weſt Indies, attracted very general notice, and ſeemingly, very deſervedly, as it apparently offers the moſt eminent and permanent advantages to ſociety. Inquiry at the Small-Pox Hoſpital, which I viſited early after my arrival in England, and reading, have convinced me of its utility; and I feel animated by a deſire, provided the Board approve it, of extending its advantages to the Magnificent, by inoculating, in a gradual and limited way, all thoſe men who have not ſuffered from Small-pox, as a ſecurity againſt its irruption at any future period. The Cow-pox, from every idea I have been able to collect relative to its nature, appears not to be communicable from the perſon inoculated to another, unleſs through the medium of inoculation; it likewiſe ſeems not to be ſtrictly a puſtular diſeaſe, of courſe not loathſome either in point of ſmell or otherwiſe; and the perſon who has been ſubjected to its action, is not only ſecured againſt its influence, but is alſo inſulated as to the power of the variolous contagion; and, to crown all theſe advantages, the fever and exceſſive excitement conſequent to its abſorption, ſcarcely merit the appellation of diſeaſe — Circumſtances recommending it in the fulleſt degree for naval practice, as the neceſſarily crouded ſituation of men on board of ſhip would render the introduction of a contagious diſeaſe, however mild, into a healthy man of war, with the view of ſuperceding the attack of one more virulent, active, and deadly, ſubject to be conſidered an unpleaſing and dangerous clue for the medical man to wind from; but the diſeaſe in queſtion is not liable to ſuch an objection. Independent of ſecuring our naval exertion from any depreſſion from Small-pox, during the time of war, there is a period approaching when ambition will be taught its limits, and we may ceaſe contending for our ſecurity and independence; it is then that this practice will ſhine forth with peculiar luſtre and advantage, and prove one great means of reſtoring rapidly the depopulation neceſſarily flowing from war. Men who have fought the battles of their country are drawn from all, and a great many from the remoteſt, parts of the three kingdoms, where the prejudices of education, religion, and ignorance, operate as injuries to ſociety, from the bulk being in ſome degree guided by ſuch principles; it therefore becomes a national and material point to gain an acceſſion of opinion in favour of this mild mode of inoculating, which ought not to be allowed to paſs unobſerved by thoſe men, provided vided it were introduced into the navy, and the reduction of prejudice, eventually arising from examples of its utility, would adhere to the mind, and, at the conclusion of the war, would prove the means of disseminating its advantages widely amongst the descendants of those who have served, and are likely to become themselves in their turn the bulwark of our country. Our commercial interests, as well as gratitude arising from the brilliancy of our naval victories, and a knowledge of the number who have fallen during the pending contest, demand the extension of a fostering hand to this order of men.

The discovery of the circulation of the blood, which for a time injured the fame and reputation of the immortal Harvey, is a wonderful example of the narrowness and perversion of ignorance; and the opposition which the introduction of the inoculation for the Small-pox met in London and throughout England, is fresh in the memory of thousands. Hence the necessity for annihilating prejudice in bringing about a revolution of so much importance, and pointing out, by every possible means, the track proper to be followed; for what gutta serena is to the eye, prejudice is to the mind.

I am in possession of the vaccine virus; but I conceive it my duty to avoid any application for its being carried into effect, until I receive the opinion of the Board, who may perceive dangers I am not aware of. I am,

Gentlemen,

Your very humble servant,

JAMES VEITEN.

